# A Prospective Cluster-Randomized Trial of Telehealth Coaching to Promote Bone Health and Nutrition in Deployed Soldiers

**DOI:** 10.3390/healthcare2040505

**Published:** 2014-12-18

**Authors:** Mary S. McCarthy

**Affiliations:** Center for Nursing Science and Clinical Inquiry, Madigan Army Medical Center, Tacoma, WA 98431, USA; E-Mail: mary.s.mccarthy1.civ@mail.mil; Tel.: +1-253-968-3695; Fax: +1-253-968-2559

**Keywords:** bone health, nutrition, vitamin D, military, deployment, telehealth coaching

## Abstract

Findings from previous studies suggest that inadequate consumption of calcium and vitamin D and a decrease in exercise while deployed can be detrimental to bone health. This study enrolled 234 soldiers randomized to receive one-time nutrition and exercise education pre-deployment (n = 149), or telehealth coaching (n = 85), throughout the deployment cycle. Results suggest that online educational efforts may enhance sports activity, bone turnover, and vitamin D status. Improving vitamin D status and remaining active while deployed appears to sustain healthy bone density in young soldiers. Early and aggressive educational outreach to young adults may prevent chronic musculoskeletal conditions and disabling osteoporosis later in life.

## 1. Introduction

According to a recent report, 2.6 million men and women have deployed in support of Operation Enduring Freedom and Operation Iraqi Freedom, and while 32,799 or more have been wounded in action, countless others will suffer physically from the “wear and tear” of war for months or years to come [1]. Annually, the Medical Surveillance Monthly Report [2] describes the morbidity burden attributable to various illnesses and injuries for all active duty service members during the previous year by medical encounters and individuals affected; of the 10 conditions that affected the greatest number of service members (over 250,000), two were musculoskeletal diseases (primarily back problems) and three were bony injuries to joints or extremities (arm, shoulder, knee, foot, ankle). The International Classification of Diseases, Ninth Revision, Clinical Modification code for musculoskeletal injuries also includes conditions such as recurrent shoulder dislocations, rotator cuff tears, stress fractures, and injury‑related cervical and lumbar strains [3]. Musculoskeletal injuries have a greater impact on the physical health and readiness of the U.S. Army than any other medical condition in peacetime or conflict, leading to limited duty, lost work days, and long-term discomfort [4]. The Army leadership strives to ensure the continuous safety, health, and performance of Soldiers in combat. To this end, proper nutrition and physical fitness remain priorities of force health protection during deployments at all levels of Command. Conditions of deployment such as heavy body armor, environmental extremes, and changes to diet and exercise habits, coupled with dehydration, fatigue, and psychological stress can lead to deterioration in the physical and mental health of young Warfighters who are expected to be at peak performance during combat. These Warfighters are predominantly enlisted soldiers who comprise 82% of the total Army force, with 69% of them between 17 and 30 years of age today [5]. This age range coincides with the period of peak bone mass, when the growth in the size of bones and the accumulation of bone mineral has stabilized. Genetic factors account for 60% to 80% of variance in peak bone mass and bone size [6] with the remainder influenced by hormonal status, diet, environmental factors, and exercise.

Deployment activities of combat arms soldiers are characterized as high intensity operations, often of a sustained nature with heavy physical demands and few rest periods. This suggests a high range of energy expenditure, fatigue, and the potential for overuse injuries. Energy expenditures of individuals in combat units range from approximately 4000 to 7131 kcal/day depending on the level of physical activity and the environment [7]. This is contrasted with garrison energy expenditures of 2500–4000 kcal/day. The excessive physical activity and associated physical stressors of deployment may result in musculoskeletal injuries, to include back pain, joint pain, and stress fractures. It is unknown if this exertional physical activity provides an opportunity to maximize bone strength or confer resistance to fractures [8]. Strong evidence supports a positive effect of mechanical loading on maintenance of bone density, but heavy loads and/or repetitive loading forces may yield muscle fatigue, whereby the capacity of muscles to protect bone from strain is compromised [9]. Load carriage requirements in the field regularly exceed 50% of lean body mass and the metabolic effects of this have not been well-studied [10]. Ten healthy male Army officers carried loads of varying weights (30%, 50%, and 70% of LBM) in an all-purpose, lightweight, individual carrying equipment pack for 30 minutes at a speed of 6 km/h. Incrementally heavier loads increased oxygen consumption, perceived exertion, and heart rate. Investigators concluded that increasing a soldier’s load requirement may negatively affect road march performance. This further supports the hypothesis that the “wear and tear” on the body has physiologic, metabolic, and musculoskeletal consequences.

In summary, the most important modifiable risk factors associated with bone density include hormonal status, physical activity, and nutrition [11,12]. Education about weight-bearing exercise and proper food choices containing calcium and vitamin D may promote strong bones, minimize stress fracture risk, and reduce musculoskeletal injuries leading to improved overall health and military readiness. The purpose of this study was to determine if telehealth coaching was superior to one-time diet and exercise education to promote nutrition status and bone health during deployment for active duty soldiers.

## 2. Experimental Section

All subjects gave their informed consent for inclusion before they participated in the study. The study was conducted in accordance with the Declaration of Helsinki, and the protocol was approved by the Ethics Committee at Madigan Army Medical Center (#122909) and the Uniformed Services University of Health Sciences (N10-C02). We used a prospective, longitudinal, cluster-randomized, controlled design with repeated measures to address calcium and vitamin D intake, physical fitness, and bone health in deployed soldiers. Volunteers were included in the study if they were 18 years of age or older, able to read and understand English, males and females in combat arms units scheduled to deploy for at least 9 months, and subjectively in good health. Exclusion criteria were diagnosis of any bone disease or current stress fractures, history of electrolyte imbalances, eating disorders, or females with diagnosed menstrual dysfunction. We recruited from a convenience sample of 250 combat arms soldiers. The first 149 soldiers enrolled comprised the Control Group (CG) who deployed first for 12 months. The second recruiting effort yielded 85 male combat arms soldiers, designated as the Telehealth Group (TG). They received online educational coaching to promote sound nutrition and bone health throughout a 9-month deployment. The use of separate units was purposeful to avoid concerns about treatment contamination.

Measurements occurred within 30 days of departure or return from the theater of operations. Once enrolled, all CG soldiers attended a one-hour educational session on healthy eating and exercise to support bone health. Following the session, baseline anthropometric measurements took place with soldiers wearing the Army physical fitness uniform of shorts, t-shirt, socks, and running shoes. Soldiers rotated through six stations which required approximately 2 hours to complete.

Height (in) was measured using a stadiometer (Seca 213, Portable Stadiometer Height Rod, Chino, CA, USA) and body weight (lbs) using a digital scale (Detecto Model DR400 electronic scale, Webb, MO, USA); no shoes were worn for these measurements. Waist circumference (WC) was obtained using a non-elastic, coated fabric measuring tape of standard length (120 in) which measured waist circumference at the minimal abdominal circumference (females) or level of the navel (males) rounded down to the nearest 0.1 in. We used the MedGem indirect calorimeter (Microlife Medical Home Solutions, Inc., Golden, CO, USA) to perform testing for resting metabolic rate (RMR). Body fat (BF)/body fat-free mass (BFFM) analysis was measured using near infrared reactance (Futrex, Inc., Hagerstown, MD, USA) and recorded as % body fat and % lean mass. Heel bone density was measured by ultrasound using a Hologic Sahara device (Foremost equipment, Rochester, NY, USA) on the dominant heel. The Hologic Sahara heel densitometer boasts the lowest ionizing radiation exposure of any competing densitometer. Results display bone mineral density with an extensive reference database of >12,000 NHANES subjects, T-score, Z-score, fracture risk assessment, and patient trending. Several studies have reported the advantages of using portable heel densitometry as it is fast, reliable, and field expedient [12,13].

A 12-item demographic tool captured relevant personal and family history related to bone health. This included the presence of any of the following: past and current use of tobacco products, alcohol consumption, medications/supplements, depomedroxyprogesterone acetate in particular, and caffeine intake, as well as age, gender, ethnicity, history of endocrine condition, history of bone disorder/stress fractures, and family history of osteoporosis.

Serum biomarkers of bone health were drawn at baseline and follow-up; markers included calcium, 25-hydroxyvitamin (25(OH) vitamin D, osteocalcin, insulin-like growth factor (IGF)-1, bone-specific alkaline phosphatase (BS alk phos), and thyroid stimulating hormone (TSH). Phlebotomy was performed by trained Army medics or nurses on site where all measurements were taken in order to ensure blood tests were processed in a timely manner. Specimens were placed in a cooler and transported to the hospital laboratory for processing every 2 hours. Very few specimens were lost to hemolysis or insufficient quantity of blood.

Dietary intake over the past year was obtained using the Block Food Frequency Questionnaire (2005.1) [14]. This questionnaire estimates usual and customary intake of a wide array of nutrients and food groups (about 110 food items). It takes 30–40 minutes to complete and is intended for self-administration. The food list for this questionnaire was developed from the NHANES 1999–2002 dietary recall data. The nutrient database was developed from the USDA Nutrient Database for Standard Reference. We purchased questionnaires from NutritionQuest (Berkeley, CA, USA) and returned them for processing which includes scanning, nutrient analysis, and computer editing. Results of the nutrient analysis, with the breakdown of all dietary components to include % carbohydrate, % fat, % protein, vitamins, minerals, and supplements consumed, were returned on a diskette along with completed questionnaires.

We acquired physical activity data using the Baecke Habitual Physical Activity Questionnaire (BHPAQ), a self-report measure of participation in habitual physical activity [15]. The BHPAQ was chosen for this study because it is short, easy to complete, and evidence for its validity and reliability have been described in a variety of populations of young adults [15,16,17,18]. The tool consists of 16 items that assess physical activity in three areas: work, sports, leisure time. The BHPAQ allows for computation of a total activity score and of specific work, sport, and leisure scores, with mean scores representing indices of physical activity in each of these areas. Anthropometric data, dietary intake, and exercise activities were obtained again, within 30 days of returning from deployment.

The telehealth coaching intervention was created as an online mode of communication to assist soldiers in the TG with access to just-in-time information. The initial website was established in the Army Knowledge Online (AKO) web portal. An alternate method established was the Army Outlook email platform which allowed secure, direct communication between participants and coaches. Access to the websites was password-protected and only soldiers in the TG were provided with a password. Posted materials and email correspondence was tailored to study aims; emphasis was on calcium and vitamin D intake from food/supplement sources, as well as bone-building exercise tips and techniques. Content experts in bone health reviewed the content of the site for accuracy and appropriateness while nurses with previous experience in using web-based health education reviewed the site for usefulness and ease of navigation. Measures of the “dose” of the intervention included number of log-ins, number of email exchanges (sent/responded), web viewing time, and tip sheets viewed/downloaded. Team members referred the participant to links on the study website to fully address each question or concern. We retained the server log to validate the number of times an individual may have logged on to the site and whether or not other individuals gained access without permission.

Descriptive statistics and relational logic checks were utilized to identify missing and invalid values within the preliminary datasets. Continuous variables from dietary intake, body composition, and physical activity changes from pre- to post-deployment were analyzed using paired *t*-tests to identify changes that occurred during deployment. For categorical variables the tests of difference in change over time were computed using generalized linear mixed models. The Multivariate Imputation by Chained Equations (MICE) with classification and regression trees was used as the underlying modeling technique. Final analyses were conducted using the MICE package [19] in the statistical software R v3.0.1. (R Foundation for Statistical Computing, 2013).

## 3. Results and Discussion

A total of 234 male and female soldiers from combat arms units on Joint Base Lewis-McChord volunteered to participate in the study. The CG, comprised of 149 soldiers who deployed for 12 months, returned first; 92 were available for post-deployment measurements. In the TG, 64 of the initial 85 soldiers were available for measurements. The overall attrition rate was 33%. This is not atypical for deployment-related soldier studies; this return rate is considerably higher than most reports [20,21]. Reasons for the attrition include reassignment to a new unit, discharge from the Army, injuries sustained during deployment with early return, or the soldier could not be located in a timely manner. We decided to exclude the data for the 14 female participants for this analysis because only one returned for post‑deployment measurements. Demographic information on participants is listed in Table 1.

**Table 1:** Demographic summary.

Variable	Telehealth Group (TG)	Control Group (CG)
Age (y)	23.87 (0.37)	23.72 (0.25)
Gender	M 100%	M 94%
Ethnicity		
Caucasian	82.3%	71.1%
Black	11.8%	18.1%
Asian	0%	2.7%
Native American/	5.9%	3.4%
Pacific Islander		
Missing	1.5%	4.7%
Race		
Hispanic/Latino	18%	13.4%
Tobacco Use		
No	43.2%	45%
Yes	52.1%	55%
Missing	4.7%	0%
Alcohol Use		
No	28.4%	28%
Yes	66.9%	72%
Missing	4.7%	0%
Stress Fracture History		
No	91.7%	85.1%
Yes	8.3%	14.9%

Reported as Mean (SD) or Frequency.

Pre- to post-deployment changes within groups and between groups in anthropometrics and bone density are shown in Table 2. After a False Discovery Rate (FDR) correction was applied for multiple testing, only change in body fat was significant within and between groups from baseline to follow up.

**Table 2:** Pre- to post-deployment changes in anthropometrics.

Variable	Pre-deployment (Baseline)		Post-deployment (Follow Up)		Change: Follow Up-Baseline		Diff	FDR
	CG (n = 135)	TG (n = 85)	CG (n = 135)	TG (n = 85)	CG (n = 135)	TG (n = 85)	*p*	*p*
Height (in)	69.4 (0.23)	69.13 (0.3)	−	−	−	−	−	−
Weight (lbs)	188.15 (2.61)	180.94 (3.51)	185.12 (2.58)	181.1 (3.24)	−3.03 (1.19)	0.16 (1.46)	0.09	0.23
Waist circ	34.33 (0.31)	33.65 (0.38)	33.79 (0.27)	33.76 (0.34)	−0.53 (0.19)	0.11 (0.24)	0.04	0.21
**Variable**	**CG (n = 135)**	**TG (n = 85)**	**CG (n = 135)**	**TG (n = 85)**	**CG (n = 135)**	**TG (n = 85)**	***p***	***p***
Body fat	19.09 (0.5)	17.44 (0.62)	18.56 (0.51)	21.38 (0.67)	−0.52 (0.42)	3.94 (0.56)	<0.0001	0.003
BMI	27.41 (0.33)	26.52 (0.41)	27.01 (0.34)	26.58 (0.42)	−0.41 (0.17)	0.06 (0.21)	0.08	0.23
RMR	2071.37 (33.53)	2016 (41.43)	1912.32 (39.8)	1954.76 (46.4)	−159.05 (45.9)	−61.24 (53.9)	0.18	0.28
BMD (gms)	0.59 (0.01)	0.62 (0.01)	0.65 (0.01)	0.64 (0.02)	0.06 (0.01)	0.02 (0.01)	0.01	0.09

Mean (SE) reported. “−“no further height measurements taken.

Select bone turnover markers are reported in Table 3. Of note is the significant change in osteocalcin across groups and across time. Osteocalcin is often referred to as a bone turnover marker because it is a secretory product of the osteoblast and is important in the bone matrix as a noncollagenous protein, and as part of the bone matrix it is released during bone resorption reflecting the cycle of turnover. It is plausible that the greater frequency and intensity of sport activity reported by the TG explains this higher level of bone turnover. Of interest is the fact that the CG demonstrated greater BMD over the course of the deployment yet work, sport, and leisure activity did not increase, and the small increases in dietary intake of protein, calcium, and vitamin D seem unlikely to impact BMD. We asked soldiers about developing stress fractures during the 9–12 months of deployment; there were 15 new stress fractures reported during the post-deployment data collection; eight in the CG and seven in the TG. This is half as many as baseline figures for the CG but about the same for the TG. It is possible that the higher levels of osteocalcin represent healing stress fractures in the TG. There is no way of knowing if participants who were lost to attrition suffered stress fractures or other musculoskeletal conditions requiring a premature return from deployment. Baseline 25(OH) vitamin D revealed a high rate of insufficiency (61%, level < 30 ng/mL) and moderate level of deficiency (17%, level < 20 ng/mL) in the total sample. Participants substantially improved their 25(OH) vitamin D levels post-deployment but only the CG had a mean value above the recommended level of 30 ng/mL.

Regarding physical activity, soldiers in the TG reported greater frequency, intensity, and proportion of time spent on sport activity during deployment than the CG resulting in a significantly higher score on the sport index of the BHPAQ (*p* = 0.015). More soldiers in the TG also reported active participation in a second sport than the CG although this was not statistically significant. This gives some indication that the telehealth communications may have been beneficial in encouraging TG soldiers to engage in weight-bearing, muscle-strengthening exercises. The most frequently reported sports included soccer, weightlifting, basketball, and running, all endorsed by the American Osteoporosis Foundation to build and maintain bone density [22]. The presence and availability of a gym or any type of exercise facility is dependent on location; the more mature bases are more likely to have gyms with a wide variety of exercise equipment and structured classes.

**Table 3:** Bone turnover markers pre- and post-deployment.

Variable	Baseline Mean (SE) CG (n = 135) TG (n = 85)		Follow Up Mean (SE) CG (n = 135) TG (n = 85)		Change: Follow Up-Baseline CG (n = 135) TG (n = 85)		*p*	FDR *p*
IGF-1	189.1 (5.25)	205.24 (6.72)	178.74 (4.74)	184.18 (6.17)	−10.36 (5.38)	−21.06 (6.89)	0.21	0.31
25(OH) Vitamin D	27.75 (0.7)	21.65 (0.86)*	34.35 (0.94)	26.02 (1.11)*	6.6 (0.82)	4.37 (0.91)	0.06	0.23
Calcium	9.59 (0.03)	9.58 (0.04)	9.45 (0.03)	9.44 (0.04)	−0.15 (0.03)	−0.14 (0.04)	0.93	0.96
BS Alk phos	15.09 (0.53)	18.73 (0.67)*	17.12 (0.72)	20.22 (0.86)*	2.02 (0.59)	1.49 (0.7)	0.56	0.67
TSH	1.66 (0.08)	1.69 (0.1)	1.88 (0.17)	1.54 (0.18)	0.21 (0.16)	−0.15 (0.16)	0.09	0.23
Osteocalcin	18.48 (0.62)	21.12 (0.77)*	22.05 (0.99)	28.31 (1.08)*	3.57 (0.77)	7.18 (0.78)*	0.001	0.01

* Denotes significance between groups at baseline, at follow up, or across time; *p* < 0.05.

Dietary intake during the time of deployment was remarkably similar for both the CG and the TG as shown in Table 4. At baseline there were very slight differences and the change from deployment and baseline were negligible with no statistically significant differences in calories, micronutrients, macronutrients, calcium, vitamin D, phosphorus, vitamin C, vitamin K, or supplemental vitamins of any kind. This may well be a reflection of the foods that were available in the dining facility during deployment which is the main, and often, the only dining option. We did not capture data specific to the dining facility menu at the various locations. Anecdotally, soldiers often reported a distaste for the milk in the dining facility and therefore many seldom drank this excellent source of calcium, vitamin D, and protein. Early in the war effort fast food trucks and establishments were allowed to exist and serve soldiers when off duty. Over time soldiers struggled to meet Army fitness standards, partly due to weight gain during deployment, so most of the fast food operations were closed or made off-limits by the ranking Commander of U.S. and International Forces in Afghanistan, then Gen. Stanley McChrystal.

Lastly, we attempted to quantify the “dose” of the coaching intervention by documenting frequency of email correspondence and downloads of the material posted online weekly. We had two sources of online information available; we learned that Outlook email was the most reliable and most likely to be read by participants. The MilBook platform was not user-friendly and therefore seldom accessed by anyone. We included a “read receipt” notification for all online postings which included brief coverage of a relevant health topic weekly and a lengthier newsletter covering several topics on two occasions. Newsletters were mailed to theater as an attempt to maintain personal contact with our TG. A small but consistent number of soldiers in the TG, ~23% of the total, corresponded with the study team. Most inquiries were related to advice about protein supplements, dietary adjustments for conditions such as lactose intolerance, questions about substitutions on the paleolithic diet or other diet plans, and calorie limits when attempting to lose weight. Soldiers reported that they had viewed many of the online postings but had not downloaded them due to lack of access to a printer. Examples of online postings include material discussing hydration, healthy servings of fruits and vegetables, how to get calcium and vitamin D into the diet, appropriate exposure to the sun, exercise routines, and limiting soda, caffeine, and tobacco use. Alcohol was prohibited in theater so this was not a concern. Internet capabilities varied greatly from one duty location to another in theater and often times a Unit went days without access to the internet. In addition, the limited time allotted for internet use meant that most soldiers were corresponding with their girlfriend, spouse, family members, and friends, and not with the research team.

**Table 4:** Dietary intake pre- and post-deployment.

Variable	Baseline		Follow Up		FDR *p*
	Control (CG)	Telehealth Group (TG)	Control (CG)	Telehealth Group (TG)	
Calories	2593.48 (112.61)	2773.89 (161.11)	2661.21 (112.95)	2537.55 (142.78)	0.249
Protein (g)	101.94 (5.04)	108.59 (6.83)	107.71 (5.14)	101.82 (6.6)	0.281
Fat (g)	102.95 (5.15)	110.81 (7.27)	106.37 (4.96)	101.58 (6.43)	0.252
Carbohydrate (g)	316.83 (16.51)	332.45 (22.06)	326.35 (18.37)	288.97 (22.06)	0.231
Calcium*	1111.11 (54.82)	1130.03 (70.61)	1142.82 (53.91)	1068.52 (65.07)	0.449
Phosphate	1670.45 (82.18)	1771.25 (117.54)	1817.6 (94.28)	1643.65 (115.42)	0.231
Sodium	4227.83 (214.09)	4442.03 (285.74)	4551.85 (211.51)	4199.16 (273.95)	0.247
Potassium	3046.29 (147.75)	3240.57 (185.69)	3221.84 (135.19)	3025.75 (168.86)	0.247
Vit C	147.41 (8.68)	166.25 (22.46)	169.3 (10.37)	141.89 (12.41)	0.213
Vit D	191.15 (11.75)	184.62 (12.88)	211.56 (14.61)	205.49 (17.03)	0.985
Vit K	137.71 (9.02)	146.64 (12.07)	176.16 (11.91)	154.3 (15.23)	0.269
Vit C (suppl)	66.88 (19.87)	35.21 (8.34)	75.16 (26.7)	79.2 (33.8)	0.449
Vit D (suppl)	69.42 (11.79)	65.55 (12.29)	116.49 (17.63)	123.53 (24.71)	0.779
Calcium (suppl)	86.6 (18.56)	98.35 (22.86)	107.97 (21.4)	114.53 (29.25)	0.953

* All electrolytes and vitamins in mg.

The study has several strengths and limitations. By focusing on wellness during deployment we were able to pique the interest of several Commanders who willingly volunteered their combat arms soldiers as potential participants. However, no individual of authority was present during the recruitment briefings and all soldiers volunteered without coercion or fear of reprisal. The choice to use separate groups under separate Commanders was intentional to avoid contamination of the telehealth coaching intervention. It would have been more challenging to respond to email inquiries quickly if the group assignment for every participant had to be checked before responding. Also, conversation amongst soldiers deployed together may have led to sharing advice from the coach which could have affected fitness behaviors of others, particularly in the CG. Another strength of the study was the highly qualified research team comprised of two credentialed dietitians interacting with the soldiers frequently, a doctorally-prepared dietitian/exercise physiologist consultant, a PhD-prepared nurse team leader, and an experienced endocrinologist, all available to address any metabolic issue that might arise. The low sample of females enrolled is most unfortunate and leaves many questions unanswered regarding their deployment health behaviors. Every effort was made to encourage soldiers to return for follow up measurements. A postcard with a reminder to return to the data collection point within 30 days of redeployment was sent to all soldiers in each group about 60 days prior to their return. A weakness of the study is the difference in the length of the deployments; we anticipated that both units would deploy for 12 months but the TG deployment occurred during the military drawdown and the time in theater was shortened to 9 months. This allowed less time for our telehealth intervention and may have disadvantaged this group in terms of exercise and dietary behaviors. This is one of the first studies on Joint Base Lewis-McChord to recruit soldiers in the unit area and perform all study measurements onsite in the unit classroom, both before and after a deployment. We feel certain that our efforts to accommodate the unit in this manner led to a more robust enrollment and low attrition rate.

## 4. Conclusions

We enrolled 234 soldiers in this prospective randomized controlled trial to evaluate telehealth options for maintaining nutrition status and bone health. We examined anthropometrics, dietary intake, physical activity, and bone biomarkers to measure the impact of internet coaching between one group of soldiers receiving on-demand health information and another group who received only one-time diet and exercise education prior to deployment. While overall impact appears low, the TG did demonstrate significantly greater bone turnover and an increase in sport activity, with greater numbers of soldiers participating in a second sport activity, although this was not significant. Bone mineral density and bone turnover were stable over time in both groups demonstrating that deployment may not have as negative an impact on bone health as previously thought. However, the TG also experienced a 4% increase in body fat while the CG experienced a modest decline. This may represent a shift to greater body fat mass and less lean mass from general changes to diet and exercise/physical training behaviors, although not evident in survey data. Body weight, waist circumference, and BMI were essentially unchanged for the TG as well. The two major brigades involved in this study deployed to Afghanistan but at different times and most likely to different locations. The availability of gyms and the appeal of the dining facilities was very site‑specific. In order to promote healthy living and morale during deployment offering similar resources and recreational options across the deployed environments seems important. Our results are similar to those reported by Lester *et al*. (2010) and their recommendation was for Commanders and unit medical personnel to encourage individual fitness in the deployed environment if unit level training is not practical [22]. We strongly concur. Our telehealth approach for coaching individuals while deployed was less effective than hypothesized as it did not reach the critical volume of participants necessary to achieve robust measurable changes.

Choices regarding lifestyle are important for all young adults but the challenges to a balanced diet and exercise regimen in the deployed environment may have longstanding consequences for the soldier, and a fit-and-ready force. Normal vitamin D status and remaining active while deployed appears to sustain healthy bone density in young soldiers. Health promotion efforts by Brigade-level public health nurses, during deployment and in peacetime, can have a major impact on lifestyle behaviors and bone health of young soldiers who are developing peak bone mass. Early and aggressive educational outreach efforts using creative technologies can help prevent chronic musculoskeletal conditions and disabling osteoporosis.
